# Exploring Nurses’ Emotional Resilience and Coping Strategies in Palliative and End-of-Life Care Settings in Saudi Arabia: A Qualitative Study

**DOI:** 10.3390/healthcare12161647

**Published:** 2024-08-19

**Authors:** Abdulaziz M. Alodhialah, Ashwaq A. Almutairi, Mohammed Almutairi

**Affiliations:** 1 Department of Medical Surgical Nursing, College of Nursing, King Saud University, Riyadh 11451, Saudi Arabia; mohalmutairi@ksu.edu.sa; 2 School of Nursing & Midwifery, Monash University, Melbourne, VIC 3800, Australia; ashwaq.almutairi@monash.edu

**Keywords:** palliative care, end-of-life care, emotional resilience, nursing, qualitative research, coping strategies, professional well-being

## Abstract

Background: Nurses working in palliative and end-of-life care settings face significant emotional challenges due to the demanding nature of their profession. This study aimed to explore the emotional resilience of these nurses, understanding the factors that contribute to their resilience, the impact on their professional well-being and performance, and strategies to enhance resilience. Methods: A qualitative study was conducted involving 15 registered nurses from various healthcare facilities in Riyadh, Saudi Arabia. Data were collected through semi-structured interviews, document analysis, and observational data. Thematic analysis was employed to identify recurring themes. Results: The study identified three key themes: Emotional Challenges and Resilience-Building, Support Systems and Resources for Resilience, and Professional Growth and Fulfillment as Resilience Factors. The findings revealed the emotional toll of patient suffering, highlighting resilience-building strategies, such as peer support, mindfulness, and reflective practices. Support systems, including workplace support, organizational resources, and mentorship, were identified as crucial for fostering resilience. Professional growth, a sense of purpose, and recognition emerged as factors contributing to resilience. Conclusions: This study underscores the importance of emotional resilience for nurses in palliative and end-of-life care settings. Healthcare organizations can enhance resilience by implementing resilience training, providing counseling services, fostering a supportive culture, and offering professional development opportunities. Addressing the emotional needs of nurses is vital for their well-being and the delivery of compassionate care.

## 1. Introduction

Nurses working in palliative and end-of-life care settings require a high level of emotional resilience due to the demanding nature of their profession [1]. These nurses offer medical assistance to patients who are dealing with life-threatening diseases and provide emotional and psychological support to their families during challenging times [2,3]. Resilience refers to the ability to maintain a stable balance when facing high emotional pressure and to recover quickly from setbacks. It encompasses a broad range of coping mechanisms and adaptive processes that enable individuals to manage stress, adversity, trauma, or any major sources of distress [4]. Resilience involves personal traits, such as optimism, self-efficacy, and effective problem-solving skills, as well as external factors, like social support and a positive work environment [5,6].

Emotional resilience, a specific aspect of resilience, is the capacity to adjust to and bounce back from emotional stress and adversity. This form of resilience is particularly crucial for nurses, especially those working in palliative care settings [7]. Emotional resilience involves not only the ability to tolerate and manage pain and distress but also the capacity to develop and flourish in the face of such challenges [8]. For nurses in palliative care, emotional resilience is essential as they frequently encounter patient suffering, death, and the emotional turmoil of patients and their families. Building and maintaining emotional resilience helps these nurses provide compassionate care while safeguarding their own mental health and well-being [9].

Nurses specializing in palliative and end-of-life care frequently encounter death, dying, and sadness, resulting in elevated levels of emotional and psychological strain [10]. Nurses who regularly face suffering and loss must engage in significant emotional labor, which entails effectively controlling their own emotions while delivering compassionate care [11]. If not properly addressed, this ongoing emotional involvement can result in burnout, compassion fatigue, and moral anguish [12]. The work atmosphere in palliative care is frequently marked by a significant level of emotional intensity. Nurses are required to manage intricate interactions with patients and families, who may be undergoing significant emotional turmoil. The inclusion of emotional support in addition to physical care increases the intricacy and requirements of the job [13,14]. In addition, nurses working in these environments frequently establish intimate connections with patients and their families, resulting in a more personal and profound experience when a patient passes away [15].

Multiple variables contribute to the establishment and sustenance of emotional resilience among nurses [16]. Personal traits, such as a positive outlook, belief in one’s abilities, and effective methods for dealing with challenges, are extremely important. Nurses who view challenges as chances for personal development rather than impossible barriers tend to have greater resilience [17]. Moreover, the presence of colleagues, friends, and family provides substantial protection against the emotional pressures faced in palliative care [18]. Resilience is also influenced by professional factors, such as workplace culture and organizational support. An atmosphere that fosters teamwork and open communication and provides access to resources like counseling services might improve resilience [19]. Emotional intelligence refers to the ability to recognize, understand, and manage one’s own emotions, as well as the emotions of others. This skill is crucial in helping nurses navigate the emotional complexities of palliative and end-of-life care settings, allowing them to provide compassionate care while maintaining their own emotional well-being [20].

The well-being and performance of nurses are strongly correlated with emotional resilience. Nurses that possess resilience are more capable of managing the pressures associated with their profession, resulting in enhanced job contentment, decreased burnout, and lower rates of staff turnover [21]. There is a positive relationship between high resilience and improved mental health outcomes, such as reduced levels of anxiety and depression [22]. Emotional resilience empowers nurses to uphold exceptional patient care standards even in demanding circumstances, hence enhancing their performance [23,24]. Nurses who possess resilience are more inclined to employ constructive coping mechanisms, such as seeking social assistance, “which refers to the emotional and practical support that nurses receive from their personal and professional networks” and prioritizing self-care. These practices can augment their capacity to deliver compassionate and efficient care [25]. Furthermore, resilience has a significant role in enhancing one’s ability to make effective decisions and solve problems, particularly in the ever-changing and uncertain context of palliative care [26].

Due to the significance of emotional resilience, multiple interventions have been suggested to assist nurses in developing and sustaining resilience. These tactics can be applied at both the individual and corporate levels [27]. Practices, such as mindfulness meditation, yoga, and relaxation exercises, can assist nurses in effectively coping with stress and developing emotional resilience. These approaches enhance a feeling of tranquility and emotional equilibrium, allowing nurses to more effectively manage the challenges of their profession [28]. Promoting the involvement of nurses in reflective practice, such as the practice of journaling or participating in debriefing sessions, enables them to effectively analyze and manage their experiences and emotions [29]. Reflective practice enables nurses to obtain profound understanding of their reactions to stress and cultivate effective approaches for handling forthcoming obstacles [30]. It is crucial to prioritize self-care activities, such as ensuring sufficient rest, nutrition, and exercise, in order to preserve both physical and emotional well-being. Nurses can enhance their ability to cope with the emotional challenges of their profession by engaging in self-care practices, which allow them to recharge and develop resilience [31].

Leadership is essential to creating an atmosphere that encourages emotional resiliency. Leaders have the ability to foster a culture that encourages transparency, deliver consistent feedback, and provide resources for managing stress and advancing professionally [32]. Promoting collaboration and camaraderie among nurses can alleviate feelings of isolation and enhance their sense of support. Regular team meetings, peer support groups, and collaborative problem solving have the potential to strengthen resilience and alleviate the emotional strain experienced by individual nurses [33]. Offering instruction in resilience-enhancing methods, emotional intelligence, and coping mechanisms can equip nurses with the necessary abilities to effectively handle stress. Resilience-focused educational programs can be incorporated into professional development activities [24]. Providing nurses with mental health resources, such as counseling services and employee assistance programs, can offer vital support during periods of emotional difficulty [34]. Organizations should actively promote the accessibility of these services and actively encourage nurses to seek assistance when necessary.

Emotional resilience is an essential aspect of nursing practice in palliative and end-of-life care settings. It empowers nurses to manage the intense emotional requirements of their job, uphold their own welfare, and provide exceptional care to patients and their families [35]. Gaining insight into the elements that contribute to resilience and employing tactics to bolster it at both the individual and organizational levels can boost the resilience of nurses and enhance their capacity to flourish in these demanding circumstances [36]. Encouraging emotional resilience is not only advantageous for the mental well-being and job fulfillment of nurses but also crucial for maintaining the standard of care in palliative and end-of-life environments [17,37].

Recent studies have significantly expanded our understanding of resilience in nursing, particularly in high-stress environments like palliative and end-of-life care. For instance, Labrague (2021) emphasizes the critical role of social support and coping behaviors in enhancing psychological resilience among healthcare workers during the COVID-19 pandemic, highlighting how peer support and structured resilience training can mitigate stress and burnout [38]. Similarly, a 2024 study underscores the importance of emotional intelligence and mindfulness practices in fostering resilience, suggesting that these strategies help nurses maintain emotional stability and effective patient care even in the face of repeated exposure to death and suffering [39]. Resilience interventions tailored to the specific needs of nurses in mental health settings significantly improved their emotional well-being and job satisfaction, offering a model that could be adapted for palliative care settings [40,41]. These recent insights underscore the necessity of integrating comprehensive resilience-building programs into nursing education and professional development to better support nurses facing the unique emotional demands of palliative and end-of-life care.

Despite existing research on emotional resilience among healthcare professionals, there is limited understanding of the specific resilience strategies used by nurses in palliative and end-of-life care settings. Most studies focus broadly on emotional resilience in nursing without delving into the unique challenges and coping mechanisms pertinent to this highly demanding and emotionally charged field. This study addresses this gap by exploring the detailed experiences and strategies of nurses working in palliative and end-of-life care, providing a deeper and more nuanced understanding of the factors that contribute to their emotional resilience and how these can be supported and enhanced by healthcare organizations.

Aim of the Study

The aim of this study is to explore the emotional resilience of nurses working in palliative and end-of-life care settings. Specifically, this study seeks to understand the factors that contribute to their resilience and the impact of resilience on their professional well-being and performance and to identify strategies that can be implemented to enhance emotional resilience among these nurses.

Research Questions

1. What are the key factors that contribute to the development and maintenance of emotional resilience among nurses in palliative and end-of-life care settings?
2. How does emotional resilience impact the professional well-being and performance of nurses working in palliative and end-of-life care environments?

## 2. Materials and Methods

### 2.1. Study Design and Participants

This descriptive qualitative design study explores the emotional resilience of nurses working in palliative and end-of-life care settings. A qualitative approach was chosen to gain a deep and nuanced understanding of the subject matter through the experiences and insights of a select group of participants. The study adhered to the Standards for Reporting Qualitative Research guidelines (SRQR) [42].

### 2.2. Setting

This study was conducted from January 2024 to March 2024. The study was conducted in various healthcare facilities in Riyadh, including both public and private hospitals and home care facilities, ensuring a comprehensive representation of different care settings. This region, known for its advanced healthcare infrastructure and diverse population, provides a rich environment for exploring the emotional resilience of nurses in palliative and end-of-life care.

### 2.3. Recruitment and Sampling

Purposive sampling was employed to select participants for this study, aiming to gather a diverse group of nurses with varying levels of experience and backgrounds. The total number of nurses was 231 nurses. The sample size of 15 was chosen based on achieving data saturation, where no new themes emerged from additional interviews. This approach is supported by qualitative research guidelines [43].

Inclusion Criteria:

Registered nurses aged 23 years or older, with a minimum of 1 year of experience in palliative and end-of-life care settings, willing to participate and provide informed consent

Exclusion Criteria:

- Nurses working in fields other than palliative and end-of-life care.

The research team sought approval from the administration of the selected healthcare facilities to introduce and explain the research concept. Potential participants were identified through discussions with nursing supervisors and staff meetings. Efforts were made to ensure diversity in the participant pool, including varying levels of experience, education, and cultural backgrounds.

Study Group:

- 10 nurses working in inpatient settings.
- 5 nurses working in home care settings.

Diversity of Participants: Participants varied in age (26–55 years), gender (10 females, 5 males), education (diploma to master’s degree), and years of experience (2–30 years). Efforts were made to ensure diversity in the participant pool, including varying levels of experience, education, and cultural backgrounds.

### 2.4. Data Collection

Interviews: Interviews were conducted by trained investigators proficient in both English and Arabic, ensuring effective communication and accurate understanding. The interview process followed a standardized protocol, which included obtaining informed consent, adhering to ethical guidelines, and maintaining confidentiality. Before each interview, participants were provided with detailed information about the study, including its purpose, procedures, potential risks, and benefits, and written informed consent was obtained. The interviews followed a semi-structured format, allowing for both consistency and flexibility, with an interview guide ensuring all relevant areas were covered while allowing participants to share their unique perspectives. Interviews lasted between 40 and 60 min and were conducted in a quiet, private setting to ensure comfort and confidentiality, with both face-to-face and telephone options available to accommodate participants’ preferences. Interviews were audio-recorded with participants’ consent, and detailed notes were taken to capture key points and non-verbal cues. The interview process adhered to ethical guidelines outlined in the Declaration of Helsinki, including respect for participants’ autonomy, privacy, and confidentiality, and participants were informed of their right to withdraw from the study at any time without penalty. After each interview, participants were offered a brief debriefing session to address any immediate concerns or questions and to clarify any ambiguous responses, ensuring the reliability and ethical integrity of the interview data collected in the study.

In-Person and Telephone Interviews: Non-verbal signals during telephone interviews were standardized by noting voice tone and pauses, and detailed note-taking was performed to capture nuances in participants’ responses.

Observational Data: To complement the interviews, observational data were gathered detailing the interactions and activities of nurses within their work environment.

- Duration and Setting: Observations were conducted over several sessions, each lasting 40 to 60 min, in various settings where nurses interacted with patients and team members.
- Focus: Observers noted verbal and non-verbal communication, the physical setup, and interaction dynamics between staff and patients.
- Recording: Detailed notes and structured forms were used to record observations systematically, providing contextual depth to the interview data.

### 2.5. Credibility of the Study

Data triangulation was achieved by combining three primary data sources to enhance the credibility and validity of the findings. This approach allowed for a comprehensive understanding of the emotional resilience of nurses in palliative and end-of-life care settings. The three data sources used were the following.

1. Semi-Structured Interviews: The core of the data collection process involved conducting semi-structured interviews with 15 registered nurses from the eastern region. These interviews provided in-depth qualitative insights into the emotional resilience encountered in their daily practice.
2. Document Analysis: A thorough review of pertinent documents was conducted to cross-verify and complement the qualitative data gathered from the interviews. The documents analyzed included nursing reports (nursing reports and care guidelines were analyzed to cross-verify and complement the qualitative data gathered from interviews, providing a broader context and understanding of the resilience strategies employed by nurses), care guidelines, institutional policies, and educational materials related to resilience and emotional support.
3. Observational Data: Observations were conducted in various settings where nurses interacted with patients and team members, focusing on routine patient care, end-of-life discussions, moments of patient distress, interprofessional collaboration, and family support. These situations were consistently observed for each respondent to ensure the comparability of data. Observers noted verbal and non-verbal communication, the physical setup, and interaction dynamics systematically using detailed notes and structured forms.

Observers documented verbal and non-verbal communication, focusing on tone, clarity, empathy, and supportiveness, as well as body language and physical gestures. Interaction dynamics were assessed, including collaboration, conflict resolution, and support within the healthcare team. Observers noted the emotional responses of nurses, such as signs of stress, compassion, frustration, and resilience, and the strategies employed to manage emotions. The physical environment was documented, including the organization of space and the availability of resources. Specific nursing activities, such as administering medication, providing physical care, and engaging in end-of-life discussions, were observed with a focus on thoroughness, compassion, and competence. Multiple observers used standardized observation forms and followed a structured protocol to ensure consistency and reliability, with regular meetings held to discuss observations and align on criteria.

### 2.6. Data Analysis

**Figure 1:**
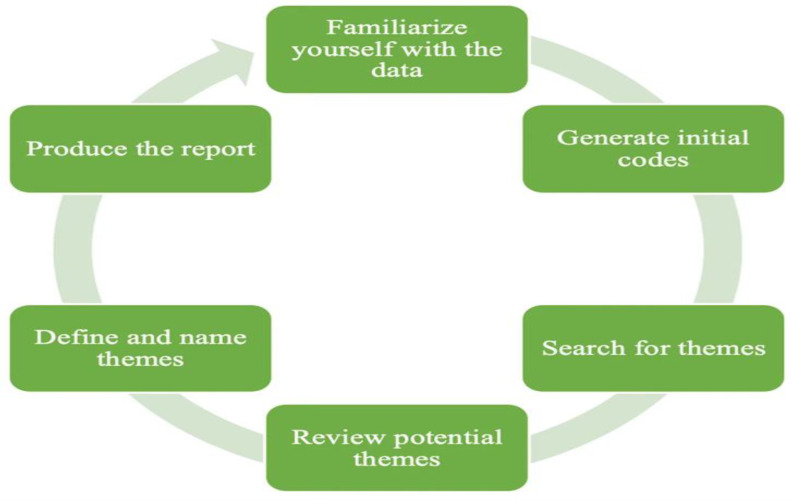
The thematic analysis’ key steps.

Data analysis followed a thematic approach as outlined by Braun and Clarke (2006). The analysis process comprised six phases (Figure 1) [44]:

1. Familiarization With the Data: Researchers read and re-read the transcripts to immerse themselves in the content.
2. Generating Initial Codes: Initial codes were generated based on recurring themes and patterns in the data.
3. Searching for Themes: Codes were organized into potential themes.
4. Reviewing Themes: Themes were reviewed and refined to ensure they accurately reflected the data.
5. Defining and Naming Themes: Clear definitions and names were assigned to each theme.
6. Producing the Report: A detailed report was generated, highlighting key findings and their implications.

Verbatim transcriptions of the interviews were generated by two trained investigators to ensure accuracy. Interviews conducted in Arabic were simultaneously translated into English by investigators fluent in both languages. An independent research assistant cross-verified selected English transcripts with their Arabic counterparts for accuracy. Thematic analysis involved an iterative process, with several researchers participating in theme identification to enhance the rigor and validity of the findings. Investigator triangulation was employed to ensure a comprehensive and nuanced understanding of the data. Multiple data analyzers reached agreement on codes and themes through an iterative process of independent coding, followed by group discussions to resolve discrepancies and achieve consensus, as recommended by Braun and Clarke (2006) [44].

### 2.7. Ethical Considerations

The study was conducted in accordance with the ethical principles outlined in the Declaration of Helsinki and approved by the ethical committee of King Saud University (KSU_HE_24_667). Informed consent was obtained from all participants prior to their inclusion in the study. Participants were informed about the study’s aims, procedures, potential risks, and benefits. Confidentiality and anonymity were assured, with data stored securely and accessible only to the research team. Measures were in place to protect participants from retribution or attrition from their supervisors. Participants involved in member-checking of interviews were informed of their right to withdraw from the study at any time without penalty.

## 3. Results

### 3.1. Participant Characteristics

Table 1 provides an overview of the 15 nurses who participated in the study. The participants exhibited a range of nursing experiences, with years of practice varying from 2 to 30 years. This spectrum includes both relatively new graduates and highly experienced practitioners, offering diverse perspectives on the emotional resilience required in palliative and end-of-life care. The participants’ age range, from 26 to 55 years, spans different stages of adult life, indicating potential generational differences in their views on emotional resilience and coping strategies. The majority of the participants are female, consistent with the long-standing prevalence of women in the nursing profession. This gender distribution reflects the broader demographics of the nursing workforce.

### 3.2. Thematic Analysis

Table 2 elaborates on the three themes identified through the thematic analysis, each reflecting the challenges and strategies related to emotional resilience faced by nurses in palliative and end-of-life care settings.

#### 3.2.1. Emotional Challenges and Resilience-Building

Regarding the extent to which each criterion affected nurses’ emotional resilience, participants described various emotional challenges they faced in their work environment, with each criterion having a distinct impact on their resilience.

- Frequency of Patient Deaths: Nurses reported that frequent patient deaths had a profound impact on their emotional resilience. One participant explained, “Dealing with multiple patient deaths in a week can be overwhelming. It sometimes makes me question my ability to continue in this field”. Another nurse shared, “Each death is a reminder of our own limitations and brings a sense of helplessness, but we must find ways to cope and move forward”. The constant exposure to death often led to emotional exhaustion and a need for effective coping strategies.
- Intensity of Suffering: The extent of suffering witnessed by nurses also significantly affected their emotional resilience. Nurses described how seeing patients in severe pain and distress on a daily basis was emotionally taxing. One participant noted, “The suffering we witness is heart-wrenching. It stays with you, even after you leave work”. Another added, “Seeing patients in pain and knowing there’s only so much we can do is one of the hardest parts of this job”. The intensity of these experiences required nurses to develop strong emotional coping mechanisms to maintain their resilience.
- Emotional Toll of Providing Palliative Care: Providing palliative care involves not only managing physical symptoms but also supporting patients and families emotionally. This dual responsibility can be emotionally draining. One nurse mentioned, “Supporting families through the loss of a loved one is emotionally exhausting. You have to be strong for them, even when you’re struggling yourself”. The emotional toll of balancing professional responsibilities with personal emotions was a common challenge faced by nurses in palliative care settings.

Extent of Suffering and Death Experienced: Nurses reported encountering an average of five patient deaths per month. The intensity of these experiences varied, but all participants acknowledged the profound emotional impact. One nurse shared, “The suffering we witness is heart-wrenching. It stays with you, even after you leave work”. Another noted, “Seeing patients in pain and knowing there’s only so much we can do is one of the hardest parts of this job”.

Coping Mechanisms: Nurses employed various coping mechanisms to deal with the emotional challenges of their work. These included mindfulness practices, seeking peer support, and engaging in reflective journaling. One nurse shared, “Mindfulness helps me stay grounded and manage my emotions better”. Another nurse mentioned, “Talking to colleagues who understand what you’re going through makes a big difference. We support each other through the toughest times”. Reflective journaling was also highlighted as a useful tool, with a participant noting, “Writing down my thoughts and feelings helps me process and release the stress”.

#### 3.2.2. Support Systems and Resources for Resilience

##### Workplace Support

Support systems and resources emerged as crucial for maintaining emotional resilience among nurses working in palliative and end-of-life care settings. Participants consistently highlighted the importance of having strong support networks within their workplace. For instance, one participant (P9) shared, “Having a supportive team makes a big difference. We rely on each other to get through tough days”. This sentiment was echoed by another participant (P6), who stated, “Our manager is very supportive, and that helps us feel valued and understood”. The sense of camaraderie and mutual support was emphasized as a key factor in helping nurses manage the emotional challenges of their work. As P11 noted, “The camaraderie among our team members is what keeps me going through the hardest times”.

##### Organizational Resources

In addition to peer support, participants pointed out the need for organizational support in the form of counseling services and resilience training. Access to these resources was seen as essential for emotional well-being. One participant (P12) mentioned, “Access to counseling services has been invaluable for me. It provides a safe space to talk about my feelings”. Another participant (P14) emphasized the benefits of resilience training, stating, “The resilience workshops provided by our hospital have taught me valuable skills to manage stress and maintain my well-being”. There was also a strong call for continuous professional development, as highlighted by P15: “Continuous professional development and stress management courses should be a regular part of our training”. These resources were identified as critical components in building and sustaining emotional resilience.

##### Mentorship

The role of mentorship was also highlighted as a significant resource for building resilience. Participants expressed the value of having experienced mentors who could provide guidance and emotional support. P5 stated, “Having a mentor who understands the challenges of palliative care has been incredibly helpful. They provide guidance and support”. This view was supported by P7, who added, “Mentorship programs should be more widely available to help new nurses navigate the emotional demands of the job”. Mentorship was seen not only as a means of professional development but also as a vital support system that helps nurses cope with the stresses of their work. As P3 remarked, “My mentor has been a pillar of support, helping me develop coping strategies and providing a sounding board for my frustrations”.

#### 3.2.3. Professional Growth and Fulfillment as a Resilience Factor

##### Sense of Purpose

Despite the emotional challenges inherent in palliative and end-of-life care, many participants found that professional growth and personal fulfillment significantly contributed to their resilience. Nurses described a profound sense of purpose and satisfaction derived from providing compassionate care to patients in their final days. One participant (P2) remarked, “Knowing that I can make a difference in a patient’s final days is incredibly rewarding”. This sentiment was echoed by another participant (P15), who said, “The bonds I form with patients and their families are special, and they remind me why I chose this profession”. Similarly, P10 added, “Every day, I feel like I am doing meaningful work that truly matters”. These reflections illustrate how a strong sense of purpose in their work helped nurses to navigate the emotional demands of their role and maintain their resilience.

##### Opportunities for Development

The sense of personal fulfillment was also closely tied to opportunities for professional development, which played a crucial role in strengthening nurses’ resilience. Continuous learning and skill enhancement were highlighted as key factors that kept participants motivated and engaged in their work. P5 noted, “The continuous learning and development opportunities keep me motivated and engaged”. Another participant (P13) emphasized the importance of professional growth, stating, “Professional growth is important. It helps me stay resilient by feeling competent and confident in my skills”. P8 shared, “Ongoing education and training are crucial. They help me stay updated and improve my practice”. These comments underscore how opportunities for professional development not only enhance nurses’ competence but also bolster their emotional resilience by reinforcing their sense of confidence and mastery in their roles.

##### Recognition and Appreciation

Another critical factor in building resilience, as identified by the participants, was the recognition and appreciation they received for their work. The acknowledgment of their efforts, whether through formal awards or simple expressions of gratitude, played a significant role in maintaining their morale and emotional well-being. P11 mentioned, “Being recognized for my hard work, whether through formal awards or simple thank-you notes, boosts my morale and resilience”. P8 echoed this sentiment, saying, “Appreciation from both colleagues and patients’ families goes a long way in maintaining my emotional resilience”. P14 added, “Acknowledgment of our efforts by the administration and patients’ families is immensely encouraging”. These insights highlight the importance of recognition and appreciation in fostering a supportive work environment that contributes to the resilience of nurses working in emotionally demanding settings

These themes underscore the complex emotional landscape that nurses navigate in palliative and end-of-life care. The insights gained from the participants highlight the need for comprehensive strategies and support systems to foster emotional resilience effectively.

These findings reveal the intricate balance nurses must maintain between coping with emotional demands and finding fulfillment in their work. The insights highlight the importance of organizational support and professional development in fostering emotional resilience among nurses in palliative and end-of-life care settings.

## 4. Discussion

The present study explored the emotional resilience of nurses working in palliative and end-of-life care settings, providing valuable insights into the challenges they face, the strategies they employ, and the support systems that foster their resilience. The findings highlight the significant emotional toll that witnessing patient suffering and death can have on nurses, underscoring the importance of emotional resilience in these demanding care environments.

### 4.1. Emotional Challenges and Resilience-Building

The emotional challenges reported by the participants resonate with the existing literature on the unique demands of palliative and end-of-life care nursing. Previous studies have identified the emotional burden associated with frequent exposure to patient suffering, loss, and grief as a significant stressor for nurses in these settings [45,46]. The emotional toll of witnessing patient suffering and death on a regular basis was described by the participants. The resilience-building strategies employed by the participants, such as seeking support from colleagues, practicing mindfulness, and engaging in reflective practices, are consistent with effective coping mechanisms identified in the literature [47]. Seeking social support from colleagues has been recognized as a crucial strategy for managing emotional distress and fostering resilience among healthcare professionals [38].

The subtheme of setting boundaries between personal and professional life resonates with the findings of Ramos-Vidal et al. [48], who highlighted the importance of maintaining work-life balance and engaging in self-care activities to prevent compassion fatigue and burnout. Establishing clear boundaries and engaging in activities outside of work can provide much-needed respite and contribute to overall emotional well-being [49].

### 4.2. Support Systems and Resources for Resilience

The study’s findings underscore the critical role of support systems and resources in fostering emotional resilience among nurses in palliative and end-of-life care settings. The importance of workplace support, including supportive teams and management, aligns with previous research emphasizing the significance of a positive organizational culture and strong social networks in promoting resilience [50,51].

The need for organizational resources, such as counseling services and resilience training, echoes the recommendations of various scholars [52]. Access to mental health support and targeted resilience interventions can equip nurses with the necessary coping strategies and emotional skills to navigate the challenges of their work effectively [24]. The role of mentorship, highlighted by the participants, is supported by the existing literature that recognizes the value of mentoring relationships in providing guidance, support, and professional development opportunities [53]. Mentorship can serve as a valuable resource for nurses, particularly in the early stages of their careers, by facilitating the transfer of knowledge and coping strategies from experienced professionals [54].

### 4.3. Professional Growth and Fulfillment as Resilience Factors

The sense of purpose and professional fulfillment reported by the participants as contributing factors to their resilience align with existing research on the motivational aspects of nursing. Studies have shown that finding meaning and purpose in one’s work can serve as a protective factor against burnout and enhance emotional resilience [21,55]. The importance of recognition and appreciation in boosting morale and resilience resonates with the work of Abdullah et al. [56], who found that perceived organizational support and recognition positively influence work engagement and well-being among nurses. This subtheme underscores the need for organizations to foster an environment of appreciation and recognition, which can contribute to the emotional resilience of their nursing staff.

The findings of this study contribute to a deeper understanding of the emotional resilience of nurses in palliative and end-of-life care settings and provide valuable insights for healthcare organizations, policymakers, and nurse educators. By recognizing the emotional challenges faced by these nurses and addressing the need for comprehensive support systems and resources, healthcare organizations can take proactive steps to promote emotional resilience and enhance the overall well-being and performance of their nursing staff [23]. This study’s findings align with and extend the existing literature on emotional resilience in nursing, particularly in the context of palliative and end-of-life care settings. The themes identified highlight the multifaceted nature of resilience and the importance of addressing individual, interpersonal, and organizational factors to foster a supportive and resilient nursing workforce.

### 4.4. Emotional Challenges and Resilience-Building

The emotional challenges described by the participants, such as the emotional toll of patient suffering and the constant need to provide emotional support to patients and families, resonate with previous research [57]. These findings underscore the intense emotional demands placed on nurses in palliative and end-of-life care settings, emphasizing the need for effective coping strategies and resilience-building interventions [12].

The resilience-building strategies employed by the participants, including seeking support from colleagues, practicing mindfulness, and engaging in reflective practices, are well-documented in the literature as effective approaches for enhancing emotional resilience [58]. The use of these strategies aligns with recommendations from organizations, such as the American Nurses Association [59], that emphasize the importance of self-care, stress management, and peer support for nurses.

The subtheme of setting boundaries between personal and professional life aligns with the concept of work–life balance, which has been recognized as a crucial factor in promoting resilience and preventing burnout [60]. By actively separating their work and personal lives, nurses can maintain a sense of balance and engage in activities that provide emotional replenishment and support their overall well-being [61].

### 4.5. Support Systems and Resources for Resilience

The findings related to the importance of workplace support, including supportive teams and management, align with existing research on the positive impact of a supportive organizational culture on nurses’ resilience [62]. A supportive work environment can foster a sense of camaraderie, open communication, and shared understanding of the emotional challenges inherent in palliative and end-of-life care nursing [63]. The need for organizational resources, such as counseling services and resilience training, reflects the growing recognition of the importance of mental health support and targeted interventions for healthcare professionals [64]. Providing nurses with access to counseling services can offer a safe space for them to process their emotions and seek professional guidance, while resilience training can equip them with practical strategies and skills to manage stress and foster emotional well-being [61].

The role of mentorship, as highlighted by the participants, is consistent with the literature on the benefits of mentoring relationships in healthcare settings [53]. Mentorship can facilitate the transfer of knowledge, skills, and coping strategies from experienced professionals to those new to the field, providing invaluable guidance and support as nurses navigate the emotional challenges of palliative and end-of-life care [54].

### 4.6. Professional Growth and Fulfillment as Resilience Factors

The sense of purpose and professional fulfillment reported by the participants as contributing factors to their resilience align with the concept of finding meaning in one’s work, which has been shown to foster resilience and mitigate the impact of stress [21]. When nurses feel that their work has a profound impact and contributes to the well-being of others, it can provide a sense of purpose and motivation that helps them persevere through challenging situations [65].

The importance of professional growth and continuous learning opportunities resonates with the literature on the role of professional development in promoting resilience [66]. By actively seeking opportunities for growth and acquiring new knowledge and skills, nurses can enhance their sense of competence and confidence, which can contribute to their emotional resilience and their ability to effectively manage the demands of their profession [24].

The subtheme of recognition and appreciation aligns with research highlighting the positive impact of perceived organizational support and recognition on work engagement and well-being among nurses [67]. When nurses feel valued and appreciated for their efforts, it can boost their morale, job satisfaction, and overall resilience, enabling them to maintain a positive outlook and continue providing high-quality care despite the emotional challenges they face [68].

The findings of this study contribute to the existing body of knowledge on emotional resilience in nursing, particularly in the context of palliative and end-of-life care settings [69]. By identifying the key factors that contribute to resilience, the impact of resilience on professional well-being and performance, and the strategies that can be implemented to enhance resilience, this study provides valuable insights for healthcare organizations, nurse educators, and policymakers [70].

Healthcare organizations can leverage these findings to develop comprehensive support systems and resources tailored to the unique needs of nurses working in palliative and end-of-life care settings [71]. This may include implementing resilience training programs, providing access to counseling services, fostering a supportive organizational culture, and establishing mentorship opportunities for nurses at various career stages [21].

Nurse educators can incorporate resilience-building strategies and emotional intelligence training into nursing curricula and continuing education programs. By equipping nurses with the necessary skills and knowledge early in their careers, they can better prepare them for the emotional challenges they may encounter in palliative and end-of-life care settings [72].

### 4.7. Emotional Challenges and Resilience-Building

The emotional challenges faced by nurses in palliative and end-of-life care settings, as described in the present study, are consistent with findings from previous research [73]. The intense emotional labor involved in providing compassionate care to patients and their families, coupled with frequent exposure to suffering and loss, can take a significant toll on nurses’ emotional well-being. These challenges underscore the critical importance of developing and maintaining emotional resilience to cope with the demands of this profession effectively [74].

The resilience-building strategies employed by the participants, such as seeking peer support, practicing mindfulness, and engaging in reflective practices, are well-documented in the literature as effective approaches for enhancing emotional resilience among healthcare professionals [74]. Peer support networks can provide a sense of shared understanding and validation, allowing nurses to process their experiences and emotions in a supportive environment [75]. Mindfulness practices have been shown to cultivate present-moment awareness, emotional regulation, and stress management skills, which are essential for maintaining resilience in demanding healthcare settings [76]. Reflective practices, such as journaling and debriefing sessions, enable nurses to gain insights into their emotional reactions and develop constructive coping mechanisms [29].

### 4.8. Support Systems and Resources for Resilience

The findings related to the importance of workplace support, including supportive teams and management, are consistent with the existing literature on the role of organizational culture in fostering resilience among nurses [77]. A positive and supportive work environment can cultivate a sense of camaraderie, open communication, and shared understanding of the emotional challenges inherent in palliative and end-of-life care nursing. When nurses feel valued, respected, and supported by their colleagues and leaders, they are better equipped to handle the emotional demands of their profession [78].

The need for organizational resources, such as counseling services and resilience training, aligns with recommendations from various professional organizations and healthcare experts [79]. Access to mental health support services can provide nurses with a confidential and safe space to process their emotions, seek guidance, and develop coping strategies. Resilience training programs can equip nurses with practical skills and techniques for managing stress, cultivating emotional intelligence, and enhancing their overall resilience [80].

#### 4.8.1. Cultural Influences on Resilience Strategies

Given the cultural context of Riyadh, Saudi Arabia, it is important to consider how local social norms and religious practices influence resilience strategies among nurses. The study’s findings suggest that community support and religious coping mechanisms are integral to the resilience-building process for many nurses in this setting. Saudi culture, which places a strong emphasis on family support and religious practices, provides a unique framework through which nurses manage the emotional challenges of their work. These cultural factors likely play a significant role in shaping the resilience strategies adopted by nurses, highlighting the need for culturally tailored interventions that align with the values and beliefs of the local population. Understanding these cultural nuances is essential for developing effective resilience-building programs that are relevant and supportive of the nurses’ cultural and social contexts.

#### 4.8.2. Critical Reflection on Limitations

While this study offers valuable insights, it is important to acknowledge its limitations. The small sample size, restricted to 15 nurses in Riyadh, may limit the generalizability of the findings to other regions or healthcare settings. Additionally, the reliance on self-reported data introduces the possibility of response bias, where participants might have presented themselves in a more favorable light. These limitations suggest that caution should be exercised in applying these findings broadly, and future research should seek to include a larger, more diverse sample to enhance the generalizability of the results. Furthermore, the cross-sectional nature of the study limits the ability to draw conclusions about the long-term effectiveness of the resilience strategies identified.

#### 4.8.3. Future Research Directions

Building on the findings and limitations of this study, future research should explore the resilience strategies of nurses across different specialties, such as oncology, to determine whether the coping mechanisms identified here are applicable in other high-stress nursing environments. Longitudinal studies would also be beneficial in assessing the long-term impact of resilience-building interventions and understanding how these strategies evolve over time. Additionally, further exploration of the role of cultural factors in shaping resilience strategies is warranted, particularly in diverse or multi-cultural settings. Such research could provide deeper insights into how resilience can be effectively supported across different cultural contexts.

## 5. Conclusions

### 5.1. Implications of the Study

The findings of this study have significant implications for healthcare organizations, nurse educators, and policymakers. By highlighting the emotional challenges faced by nurses in palliative and end-of-life care settings and identifying the factors that contribute to their emotional resilience, this study provides a roadmap for developing comprehensive support systems and resources. Healthcare organizations can use these insights to implement targeted interventions, such as resilience training programs, counseling services, and mentorship opportunities, to foster a more resilient nursing workforce. Nurse educators can integrate resilience-building strategies and emotional intelligence training into nursing curricula, better preparing future nurses for the emotional demands of their profession. Policymakers can leverage these findings to develop policies and guidelines that prioritize the emotional well-being of nurses, allocating resources for mental health support services and promoting workplace initiatives that cultivate a supportive organizational culture.

### 5.2. Limitations of the Study

While this study provides valuable insights, it is essential to acknowledge its limitations. The sample size of 15 participants, although sufficient for a qualitative study, may not fully capture the diversity of experiences and perspectives within the broader population of nurses working in palliative and end-of-life care settings. Additionally, the study was conducted in a specific geographic region, and the findings may not be generalizable to other cultural or healthcare contexts. Furthermore, the study relied on self-reported data from participants, which may be subject to biases or limitations in self-awareness. Future research could employ a larger sample size and include a more diverse range of participants from various geographic regions and cultural backgrounds to enhance the generalizability of the findings.

### 5.3. Conclusions

This study provides a comprehensive examination of the emotional resilience of nurses working in palliative and end-of-life care settings in Riyadh, Saudi Arabia. The findings offer valuable insights into how workplace support, professional growth, and cultural factors contribute to resilience among these healthcare professionals. Based on the results, the following conclusions are drawn.

1. Implications for Healthcare Organizations

This study highlights the crucial role of workplace support systems in fostering resilience among palliative care nurses. Healthcare organizations are encouraged to implement and strengthen support mechanisms, such as access to counseling services, peer support programs, and effective management practices. These measures can help mitigate the emotional toll of working in palliative care settings and enhance job satisfaction and retention.

2. Importance of Professional Development

Professional growth is a significant factor in building resilience. Providing nurses with opportunities for continuous education, resilience training, and professional development is essential for maintaining their emotional well-being. Such initiatives not only improve clinical skills but also reinforce a sense of purpose and fulfillment in their roles. Healthcare institutions should prioritize these aspects as part of their strategic planning for workforce development.

3. Need for Culturally Tailored Interventions

This study underscores the impact of cultural factors on resilience strategies. In the context of Riyadh, Saudi Arabia, local cultural and religious practices play a significant role in shaping how nurses cope with the challenges of palliative care. Developing culturally tailored resilience programs that align with the values and beliefs of the local population is crucial. Future research should explore how cultural nuances influence resilience and inform the design of interventions that are both effective and culturally relevant.

#### 5.3.1. Future Research Directions

To build on the findings of this study, future research should focus on several areas:

- Expanding the Sample Size and Scope: Conduct studies with larger and more diverse samples to enhance the generalizability of the findings.
- Longitudinal Studies: Explore the long-term effects of resilience-building interventions and how resilience strategies evolve over time.
- Cultural Factors: Further investigate the role of cultural and religious factors in shaping resilience strategies in different healthcare settings.

By addressing these areas, future research can provide deeper insights into how best to support nurses in various palliative care contexts and contribute to the development of more effective and culturally sensitive resilience programs.

#### 5.3.2. Final Thoughts

In conclusion, this study reinforces the importance of supporting the emotional resilience of nurses in palliative and end-of-life care settings. By addressing workplace support, investing in professional development, and considering cultural factors, healthcare organizations can significantly enhance the well-being of their nursing staff and improve the quality of care provided to patients in these critical settings.

## Figures and Tables

**Table 1:** Participant characteristics (n = 15).

Participant ID	Age	Gender	Education	Years of Experience
P1	28	Female	Bachelor’s	5
P2	35	Male	Diploma	12
P3	42	Female	Bachelor’s	18
P4	50	Female	Bachelor’s	25
P5	30	Female	Diploma	7
P6	37	Male	Bachelor’s	13
P7	29	Female	Bachelor’s	6
P8	44	Female	Master’s	20
P9	39	Male	Bachelor’s	15
P10	33	Female	Bachelor’s	9
P11	31	Female	Diploma	8
P12	47	Female	Bachelor’s	22
P13	55	Female	Bachelor’s	30
P14	26	Male	Bachelor’s	2
P15	41	Female	Master’s	17

**Table 2:** Thematic results.

Theme	Subtheme	Description
Emotional Challenges and Resilience-Building	Frequency of Patient Deaths	Frequent patient deaths significantly impacted nurses’ emotional resilience, leading to feelings of helplessness and the need for effective coping strategies.
	Intensity of Suffering	The emotional toll of witnessing patients’ suffering daily required nurses to develop strong emotional coping mechanisms to maintain their resilience.
	Emotional Toll of Providing Palliative Care	Balancing professional responsibilities with the emotional demands of supporting patients and families was a significant challenge, leading to emotional exhaustion.
Support Systems and Resources for Resilience	Workplace Support	Strong support networks within the workplace, including camaraderie among team members and supportive management, were crucial in helping nurses manage the emotional challenges of their work.
	Organizational Resources	Access to organizational resources, such as counseling services, resilience training, and continuous professional development, were essential for maintaining emotional well-being and building resilience.
	Mentorship	Mentorship provided guidance and emotional support, helping nurses develop coping strategies and navigate the emotional demands of palliative care, particularly for less-experienced staff.
Professional Growth and Fulfillment as a Resilience Factor	Sense of Purpose	The sense of purpose and satisfaction derived from providing compassionate care significantly contributed to nurses’ resilience, helping them navigate the emotional demands of their role.
	Opportunities for Development	Continuous learning and professional growth opportunities reinforced nurses’ competence and confidence, which in turn bolstered their emotional resilience.
	Recognition and Appreciation	Recognition and appreciation from colleagues, patients’ families, and management played a significant role in maintaining nurses’ morale and emotional well-being, contributing to their resilience in emotionally demanding settings.

## Data Availability

All data are available within the manuscript.
